# Enhanced Skin Permeation of Diclofenac Sodium Using Mango Seed Kernel Starch Nanoparticles

**DOI:** 10.3390/ph18101585

**Published:** 2025-10-20

**Authors:** Sesha Rajeswari Talluri, Namrata S. Matharoo, Nirali Dholaria, Nubul Albayati, Shali John, Bozena Michniak-Kohn

**Affiliations:** 1 Ernest Mario School of Pharmacy, Rutgers-The State University of New Jersey, 160 Frelinghuysen Road, Piscataway, NJ 08854, USA; sr1798@scarletmail.rutgers.edu (S.R.T.); nsm112@scarletmail.rutgers.edu (N.S.M.); nvd16@scarletmail.rutgers.edu (N.D.); na501@gsbs.rutgers.edu (N.A.); 2 Center for Dermal Research, Rutgers-The State University of New Jersey, 145 Bevier Road, Piscataway, NJ 08854, USA; koonthsh@scarletmail.rutgers.edu

**Keywords:** mango seed kernel starch, starch nanoparticles, transdermal permeation, diclofenac sodium, agro-industrial waste

## Abstract

**Background:** Mango seed kernels, an agro-industrial waste byproduct, constitute approximately 40–50% of the fruit’s weight and serve as a substantial source of starch. There are only a few reported studies on the pharmaceutical applications of Mango Seed Kernel Starch (MSKS) and drug carriers produced from this source. This study aims to isolate starch from mango seed kernels (MSKS), prepare drug-loaded mango seed kernel starch nanoparticles (MSKSNPs), and study the in vitro transdermal permeation. **Methods:** The MSKS was prepared using the alkaline method and freeze-dried. The prepared starch was analyzed for physicochemical properties relative to corn starch. The mango seed kernel starch nanoparticles (MSKSNPs) were prepared using mild alkali hydrolysis and the ultrasonication method. The model drug selected for this study was diclofenac sodium (DS), a commonly prescribed non-steroidal anti-inflammatory drug. **Results:** The average particle size of the drug-loaded nanoparticles was 140.0 ± 3.6 nm, with a PDI of 0.42 ± 0.03. The Transmission Electron Microscopy images confirmed the globular structure of MSKSNPs. X-ray Diffraction revealed that the diclofenac crystal size decreased to 14 nm from 33 nm in the pure drug, confirming the amorphous nature of MSKSNPs. The drug-loaded MSKSNPs showed a % encapsulation efficiency of 92.4 ± 3.7 and % drug loading of 31.08 ± 0.96. The cumulative drug released from MSKSNPs after 6 h, 12 h, and 24 h was found to be 25.58 ± 1.30, 59.68 ± 2.98, and 127.5 ± 6.4 μg/cm^2,^ respectively, which was more than the ethanolic drug solution with statistical significance (*p*-value < 0.01) along with enhanced skin retention. **Conclusions:** MSKSNPs were efficiently synthesized using mild alkali hydrolysis and ultrasonication, showing enhanced transdermal delivery. Skin retention was significantly higher in MSKSNPs (*p*-value < 0.05). The cytotoxic studies revealed that both formulations exhibit similar dose-dependent cytotoxicity, with no significant difference (*p* > 0.05) in their potency under the tested conditions.

## 1. Introduction

Natural polysaccharides have become a focus of interest for many researchers today due to their diverse functions in drug delivery, with starch being one of the most prominent [1]. In addition, the pharmaceutical sector has shown a strong inclination toward these naturally derived polymers, driving significant interest in the discovery, extraction, and purification of plant-based polymers for pharmaceutical use. Starch is among the leading natural polymers in this field [2].

Starch is a polysaccharide made up of amylopectin and amylose and has various applications across various industries, including textiles, cosmetics, food, and pharmaceuticals. In the pharmaceutical industry, starch is highly versatile, serving as a binder, diluent, lubricant, and disintegrant in numerous solid dosage forms [3]. Its popularity is due to its wide availability as well as its nontoxic, biodegradable, and economical properties. Considerable research has focused on the properties of commercially important starches obtained from seeds (corn, wheat, and rice) and tubers or roots (potato, cassava, and yams), largely due to their ready availability [4]. Starches from various sources exhibit differences in morphological, structural, and physicochemical properties. These diverse applications of starch require specific functional characteristics. However, there is limited information in the literature regarding the isolation and properties of starches from non-conventional sources, such as fruit (litchi, jackfruit, avocado, pea, banana, and chestnut) and cereals (quinoa and sago) [5].

Mango (*Mangifera indica*) is a widely cultivated tropical fruit, especially in India, Thailand, and Malaysia. Agro-industries commonly use it to produce juice, jams, and nutritional supplements, with the pulp being utilized while the seeds and peels are discarded as waste. The kernels are typically thrown away when consumed fresh, even though they make up about 40–50% of the fruit’s total weight. These kernels are a rich starch source, with research indicating a 50–60% starch yield from mango seed kernels. As a result, this agricultural waste product offers a reliable and abundant starch source, presenting the potential for added value [6].

Natural starch lacks suitable stability despite its many advantages due to microbial contamination, thermal decomposition, and retrogradation. Its low mucoadhesiveness, solubility only in cold water, and weak mechanical properties, such as limited flexibility and tensile strength, restrict its use in the pharmaceutical industry [7]. To address these limitations, researchers have focused on chemically modifying starch, with techniques like physical, chemical, or enzymatic treatment improving its physicochemical properties [8]. Starch nanoparticles are one such result of these modifications [9,10].

This study aimed to prepare and characterize drug-loaded nanoparticles derived from starch isolated from mango seed kernels. These nanoparticles were further tested for in vitro transdermal permeation. Diclofenac, a widely used non-steroidal anti-inflammatory drug (NSAID), was selected as the model drug due to its suitability for developing sustained or controlled-release products. Diclofenac sodium is a very well-established drug in transdermal formulations; its physicochemical properties, such as its moderate molecular weight (~296.15 g/mol), log P (4.5), and sufficient skin permeability make it an ideal candidate for evaluating the performance of novel transdermal formulations. In studies conducted by El-Naggar et al., the authors utilized diclofenac sodium for the transdermal delivery of cross-linked starch nanoparticles [11].

There are only a few studies on the pharmaceutical applications of Mango Seed Kernel Starch (MSKS) and drug carriers from this source. The study conducted by Chaksmithanont et al. focused on optimizing starch derived from *Mangifera indica* (mango) fruit, which was modified through acid hydrolysis and pregelatinization using computer-assisted techniques to develop a potential alternative to conventional pharmaceutical tableting excipients. The optimized pre-gelatinized starch demonstrated significant improvements in flowability and compressibility, with swelling capacity increased by 3.95-fold compared to unmodified starch and 1.24-fold compared to sodium starch glycolate. Moreover, the pre-gelatinized starch exhibited a binding effect comparable to standard excipients, whereas the hydrolyzed starch showed reduced binding capacity due to its shorter polymer chains [12]. In a study by Devi et al., fast-dissolving tablets of telmisartan were developed using MSKS as a super disintegrant. The results demonstrated that MSKS possessed excellent disintegration efficiency and exhibited fine, free-flowing, and amorphous characteristics. The optimized formulation containing 5% MSKS as a super disintegrant showed the highest percentage of drug dissolved within 10 min (PD_10_) and the shortest disintegration time compared with formulations prepared using commercially available super disintegrants [13].

These studies highlight the potential of MSKS as a pharmaceutical excipient, derived from agro-industrial waste, making it a readily available and cost-effective alternative.

## 2. Results

### 2.1. Isolation and Characterization of Mango Seed Kernel Starch

The percentage yield of MSKS varied with the soild–solvent ratio and drying method (Table 1). The maximum yield was obtained with a 1:14 soild–solvent ratio. The freeze drying method yielded better granules than air drying or oven drying. The aqueous solubility of MSKS was 17.0 ± 2.8%, with a pH of 7.0 ± 1.2, moisture content of 7.4 ± 0.8%, water holding capacity of 79.35 ± 0.80%, swelling power of 3.20 ± 0.16 g/g, and gelatinization temperature of 60 °C (Table 2). Particle size was found to be 6.5 ± 0.1 μm with a polydispersity index of 0.70 ± 0.00. FTIR confirmed the chemical integrity of starch.

### 2.2. Characterization of Mango Seed Kernel Starch Nanoparticles

#### 2.2.1. Particle Size Distribution, Zeta Potential, % Encapsulation Efficiency (%EE) and % Drug Loading (%DL)

**Figure 1:**
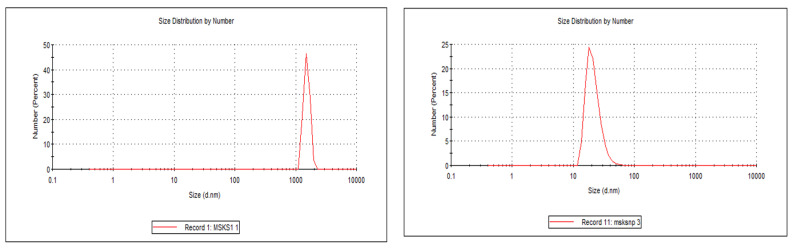
Particle size distribution of mango seed kernel starch (MSKS) on the left-hand side and mango seed kernel starch nanoparticles (MSKSNPs) on the right-hand side.

The particle size of the MSKS was found to be 6.5 ± 0.1 μm with a polydispersity index of 0.70 ± 0.0. The particle size of the MSKS was found to be 6.5 ± 0.1 μm with a polydispersity index of 0.70 ± 0.0 (Figure 1). Formulation F1, with a 1:1 ratio, exhibited a particle size of 167.0 ± 1.3 nm, a PDI of 0.34 ± 0.05, a zeta potential of −11.00 ± 0.05 mV, a % encapsulation efficiency of 83.0 ± 5.0, and a % drug loading of 28.4 ± 3.6. Formulation F2, with a 2:1 ratio, showed a reduced particle size of 140.0 ± 3.6 nm, a slightly higher PdI of 0.42 ± 0.03, an improved zeta potential of −31.20 ± 0.13 mV, the highest encapsulation efficiency of 92.4% and a % drug loading of 31.0 ± 2.0. Lastly, formulation F3, with a 1:2 ratio, achieved the smallest particle size of 123 ± 4.2 nm, the highest PdI of 0.47 ± 0.02, a zeta potential of −27.93 ± 0.14 mV, and a % encapsulation efficiency of 87.6 ± 3.0 with % drug loading of 24.0 ± 1.4 (Table 3).

#### 2.2.2. X-ray Diffraction (XRD)

**Figure 2:**
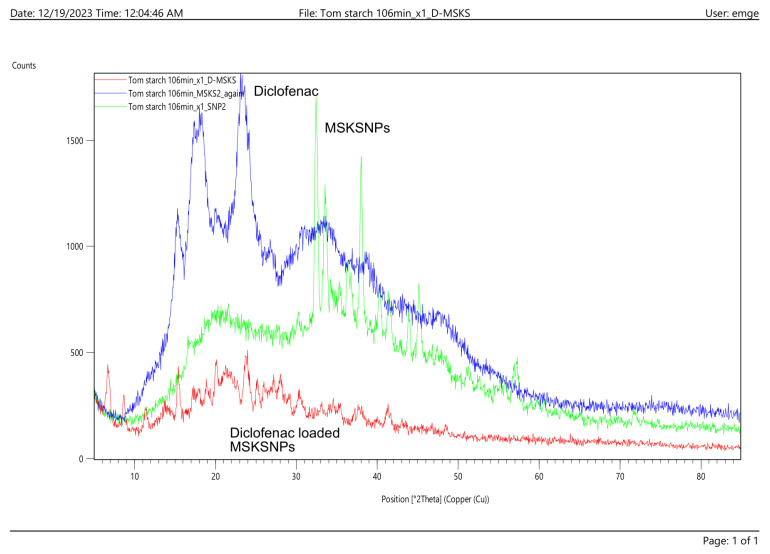
XRD pattern for diclofenac-loaded MSKSNPs and diclofenac sodium alone showing reduced crystallinity of drug-loaded mango seed kernel starch nanoparticles compared with that of diclofenac.

The X-ray diffraction analysis revealed a significant reduction in the crystal size of diclofenac in the MSKSNP formulation, with a calculated crystal size of 14 nm, compared to the 33 nm crystal size of the pure drug. This reduction in crystal size suggests the transformation of diclofenac from its crystalline form into a more amorphous state within the nanoparticles. The smaller crystal size observed in the MSKSNPs indicates a higher degree of amorphization, consistent with the characteristic features of amorphous materials, such as enhanced solubility and dissolution rate. This transformation confirms the successful incorporation of diclofenac into the MSKS nanoparticle system, facilitating improved drug delivery characteristics (Figure 2).

#### 2.2.3. Transmission Electron Microscopy (TEM)

**Figure 3:**
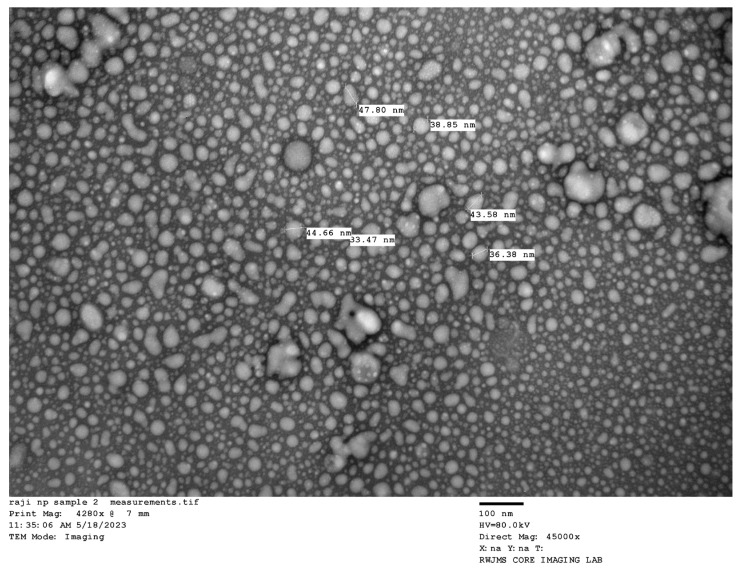
TEM image of mango seed kernel starch nanoparticles (MSKSNPs) showing globular nanoparticles with particle size < 100 nm.

This result further supports the successful fabrication of MSKSNPs at the nanoscale, confirming their uniform shape and size distribution (Figure 3).

#### 2.2.4. FTIR

**Figure 4:**
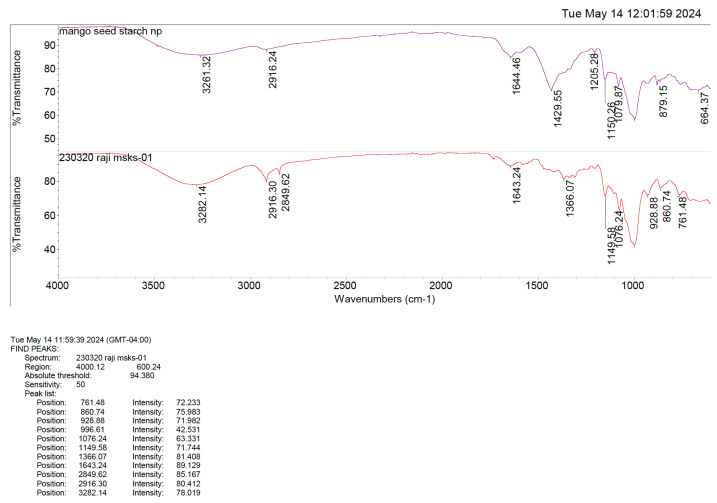
FTIR of mango seed kernel starch (MSKS) and mango seed kernel starch nanoparticles (MSKSNPs) showing the stretching of -OH bonds, indicating the hydrolysis of starch.

The Fourier-transform infrared (FTIR) spectra of the MSKSNPs revealed a strong absorption peak between 3280 and 3320 cm^−1^, which was attributed to the -OH stretching vibration. This characteristic peak is commonly associated with hydroxyl groups in starch, and its prominence indicates a significant interaction with water molecules (Figure 4).

### 2.3. In Vitro Release Studies

Drug content was determined using the HPLC method outlined in Section 4.4.2. The method achieved a retention time of 4.4 min and demonstrated excellent linearity, with a regression coefficient (R^2^) of 1 across the concentration range of 0.24–125 µg/mL. Method precision was confirmed by low intra-day and inter-day %RSD values of 0.56 ± 0.01 and 0.49 ± 0.03, respectively. The limits of detection (LOD) and quantification (LOQ) were established at 0.12 µg/mL and 0.24 µg/mL, respectively.

**Figure 5:**
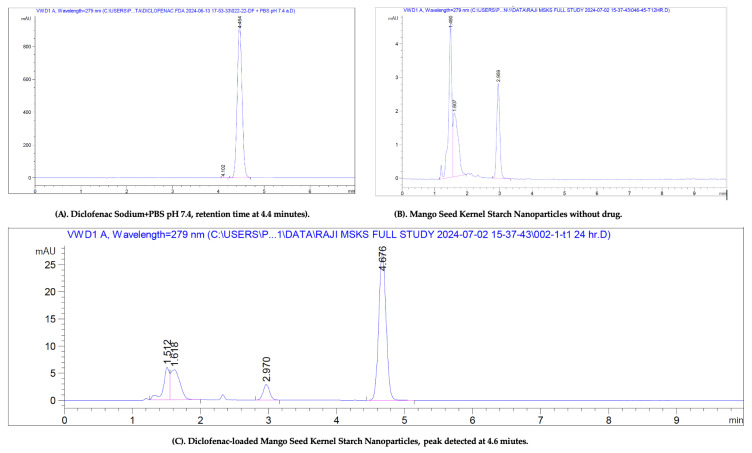
(**A**) HPLC chromatogram of diclofenac sodium in phosphate-buffered saline showing the retention time at 4.4 min with a sharp peak with no intervention. (**B**) HPLC chromatogram of mango seed kernel starch nanoparticles (MSKSNPs) without diclofenac showing no peak at 4.4 min. (**C**) Diclofenac sodium-loaded MSKSNPs showing the peak for diclofenac at 4.4 min.

The specificity of the HPLC method was evaluated by comparing chromatograms of the pure diclofenac standard, the blank formulation (without drug), and the drug-loaded formulation. As shown in Figure 5, the retention time of diclofenac was consistent across all samples, and no peaks causing interference were observed in the blank formulation, confirming the specificity of the method.

**Figure 6:**
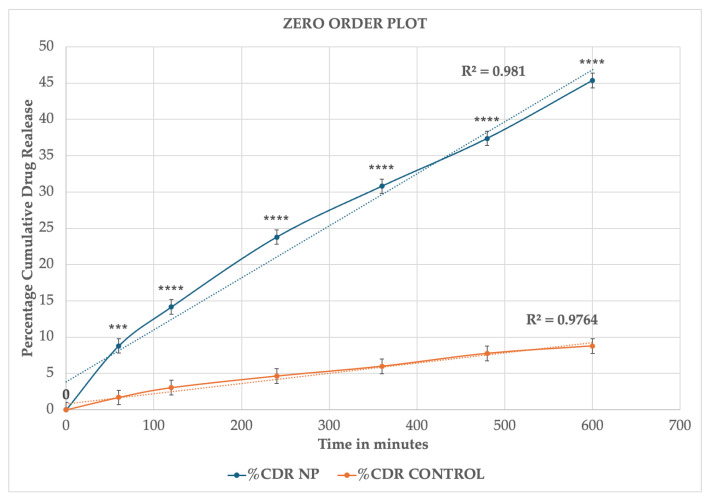
Zero-order release kinetics of diclofenac sodium from drug-loaded nanoparticles and control (ethanolic drug solution). The plot represents the percentage cumulative drug release (%CDR) versus time (minutes). The release profile of nanoparticles (%CDR NP) demonstrated a higher correlation coefficient (R^2^ = 0.981) compared to the control formulation (R^2^ = 0.9764), indicating a better fit to zero-order kinetics. Data are presented as the mean ± SD (*n* = 3). Statistical significance compared to control: *** *p* < 0.001; **** *p* < 0.0001.

**Figure 7:**
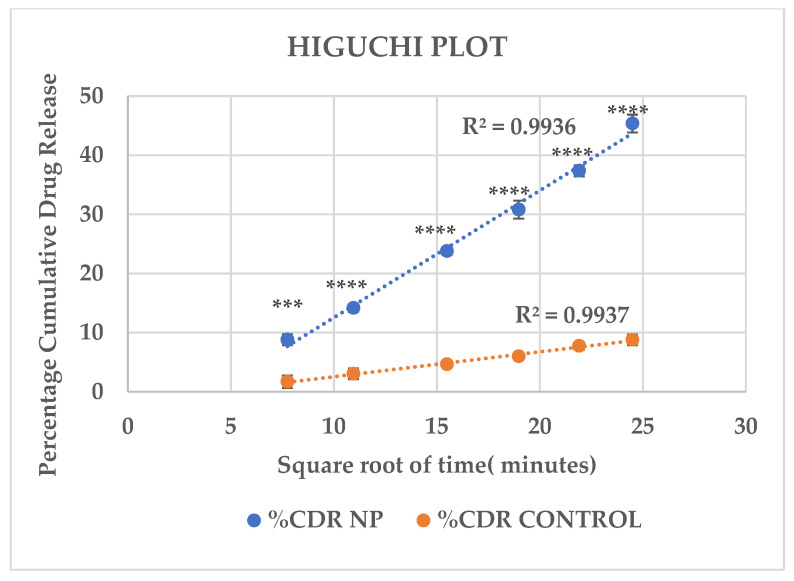
Drug release kinetics according to the Higuchi model. The plot shows the percentage of cumulative drug released (CDR) from a nanoparticle (NP) formulation and a control formulation over time. A linear correlation was observed between cumulative drug release and the square root of time indicated by the highest R^2^ values (>0.99), suggesting that the primary mechanism of drug release is diffusion-based. Asterisks denote a statistically significant variation in release among the NP and control at indicated time intervals (*** *p* < 0.001; **** *p* < 0.0001).

**Figure 8:**
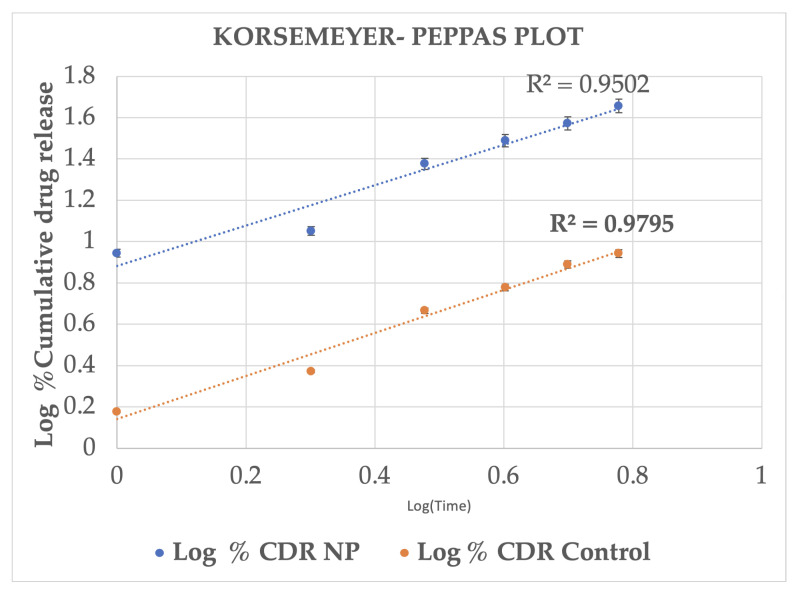
The logarithmic plot of cumulative drug release versus time is presented for the nanoparticle and control formulations. Data points represent the M ± SD. The data were fitted to the Korsmeyer–Peppas model, yielding correlation coefficients of R^2^ = 0.9502 for the NP formulation and R^2^ = 0.9795 for the control. The lower R^2^ values, particularly for the NP formulation, suggest it provides a less descriptive fit to the experimental data compared to the Higuchi model (Figure 1). Statistical analysis indicated a significant difference (*p* < 0.05) in cumulative release between the two formulations at all time points.

**Figure 9:**
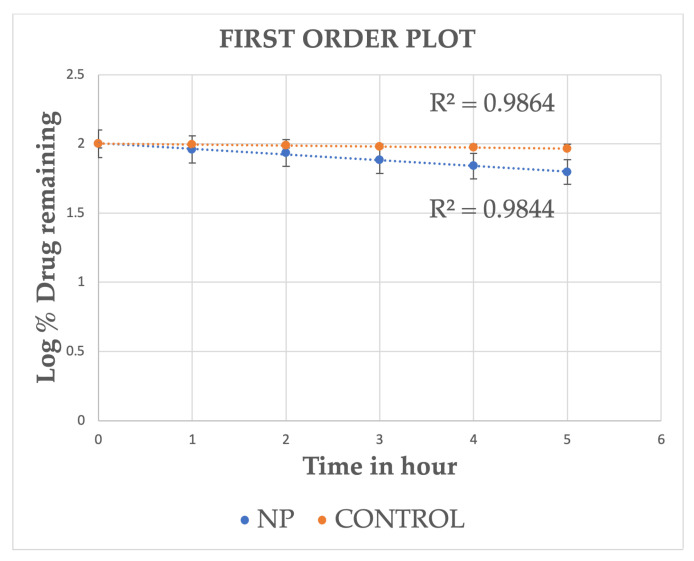
The logarithmic plot of cumulative drug remaining versus time is presented for the nanoparticle and control formulations. Data points represent the (M ± SD). The data were fitted to the first-order model, yielding correlation coefficients of R^2^ = 0.986 for the NP formulation and R^2^ = 0.984 for the control, which are lower than the Higuchi model.

The cumulative drug release profiles of drug-loaded MSKSNPs and control (Ethanolic drug solution) are shown(Table 4). The in vitro release data of both the formulations were analyzed by fitting the release profiles to various kinetic models to elucidate the release behavior (Figure 6, Figure 7, Figure 8 and Figure 9). The release profiles of both formulations conform to Higuchi’s model with the highest correlation coefficient (R^2^ values) (Table 5).

### 2.4. In Vitro Permeation Studies

**Figure 10:**
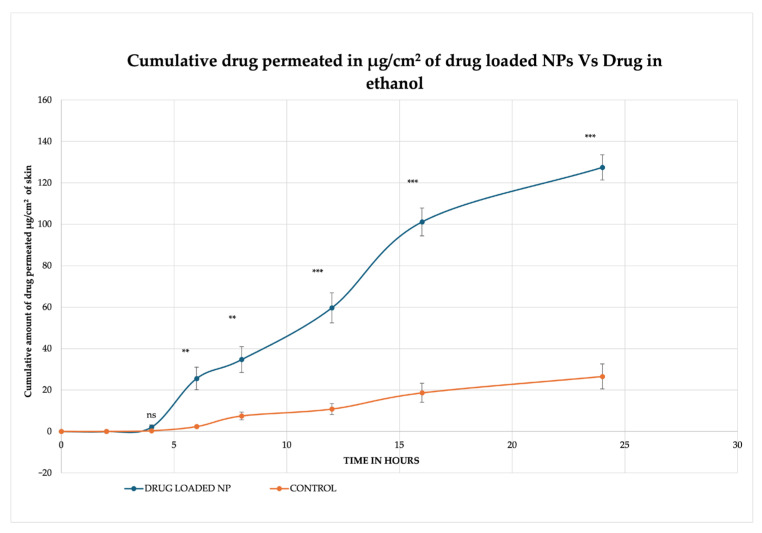
In vitro skin permeation profile of drug-loaded nanoparticles versus a control solution. The graph shows the cumulative amount of drug permeated through a skin membrane (in µg/cm^2^) over 24 h. The drug-loaded nanoparticle (NP) formulation is compared to a control solution of the drug in ethanol. Each data point reflects the (Mean ± SD). The results demonstrate that the NP formulation significantly enhanced the permeation of the drug via skin compared to the control. Statistical significance is indicated as follows: ns (not significant, *p* > 0.05), ** (*p* ≤ 0.01), and *** (*p* ≤ 0.001).

**Figure 11:**
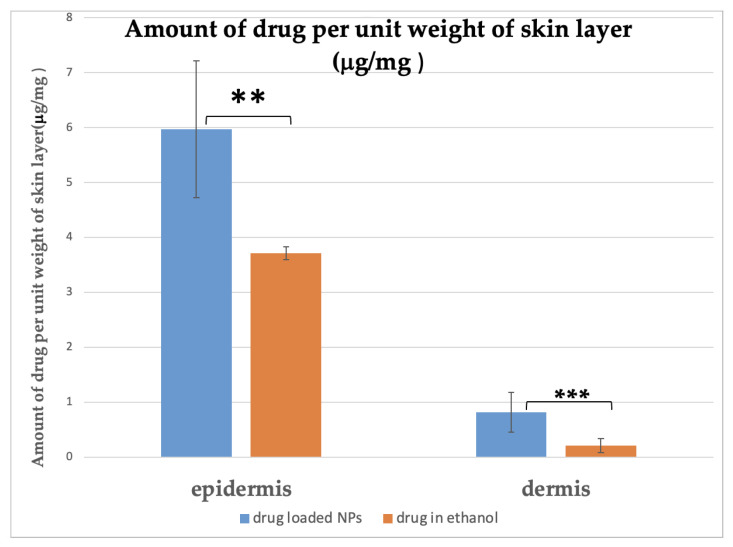
Drug deposition within epidermal and dermal skin layers. The bar chart quantifies the amount of drug retained per unit weight of tissue (µg/mg) in the epidermis and dermis at the conclusion of the ex vivo permeation study. The drug-loaded nanoparticle (NP) formulation is compared to a control solution (drug in ethanol). The results show that the NP formulation led to significantly higher drug deposition in both the epidermis (** *p* < 0.01) and the dermis (*** *p* < 0.001) compared with the control. Data corresponds to the (mean ± sd).

The cumulative amount of drug released from MSKSNPs after 4, 6, 8, 12, 16, and 24 h was found to be 2.08 ± 1.08 μg/cm^2^, 25.58 ± 1.30 μg/cm^2^, 34.62 ± 6.19 μg/cm^2^, 59.68 ± 2.98 μg/cm^2^, 101.17 ± 6.12μg/cm^2^, and 127.5 ± 6.4 μg/cm^2^ respectively. In comparison, the ethanolic solution of diclofenac sodium released significantly lower amounts of the drug: 0.35 ± 0.27 µg/cm^2^ (*p*-value > 0.05), 2.36 ± 0.11 µg/cm^2^ (*p*-value < 0.01), 7.5 ± 1.84 µg/cm^2^ (*p*-value < 0.01), 10.83 ± 0.54 µg/cm^2^ (*p*-value < 0.001), 18.63 ± 4.61 μg/cm^2^ (*p*-value < 0.001), and 26.56 ± 1.32 µg/cm^2^ (*p*-value < 0.001), at the same time intervals (Figure 10). Furthermore, the amount of diclofenac sodium found in the epidermis was higher for MSKSNPs (5.97 ± 0.30 µg/mg) compared to the ethanolic solution (3.70 ± 0.19 µg/mg) (*p* < 0.05), indicating improved skin penetration. Similarly, the drug concentration in the dermis was greater for MSKSNPs (0.82 ± 0.04 µg/mg) than the ethanolic solution (0.21 ± 0.01 µg/mg) (*p* < 0.005), highlighting the enhanced transdermal delivery of diclofenac sodium when encapsulated in MSKSNPs (Figure 11).

### 2.5. Cytotoxicity Studies

**Figure 12:**
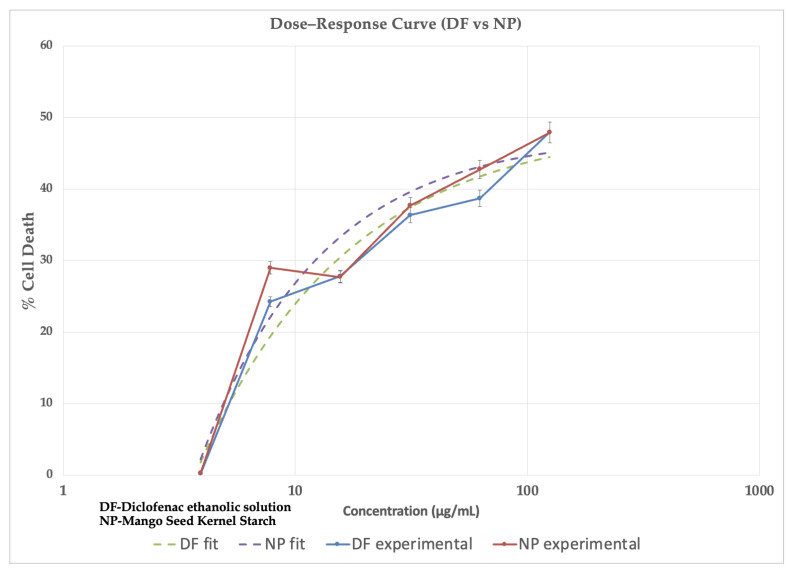
Dose–response curve comparing the cytotoxicity of drug-loaded nanoparticles (NP) against the control drug dissolved in ethanol (DF). The experimental data and the corresponding non-linear regression fits are shown for both the NP formulation and the DF control. The results indicate that both formulations exhibit similar dose-dependent cytotoxicity, with no significant difference (*p* > 0.05) in their potency under the tested conditions.

Both control and drug-loaded nanoparticles induced cell viability that declined in a concentration-dependent manner, with peak cell death observed at 47.92 ± 2.5 for the control and 47.91 ± 2.7 at 125 µg/mL (Table 6). The four-parameter logistic fit yielded IC_50_ values of 9.3 × 10^−5^ µg/mL (control) and 5.9 × 10^−4^ µg/mL (drug-loaded nanoparticles) (Table 7). The fitted top values were 48% for DF and NP formulations, consistent with the experimental maximum response(Figure 12).

## 3. Discussion

The isolation process of MSKS was significantly influenced by both the solid-to-solvent ratio and the drying method employed. Among the tested conditions, a 1:14 solid-to-solvent ratio produced the highest starch yield, indicating that an excess of solvent facilitates efficient leaching and separation of starch from non-starch components.

In terms of drying methods, freeze-drying produced superior starch granules compared to conventional air or oven drying. Freeze-drying likely preserved granule integrity and prevented structural collapse or surface damage, a common issue in oven-dried starches due to high thermal exposure [14]. The physicochemical characteristics of MSKS revealed that it possesses favorable characteristics comparable to corn starch. FTIR analysis confirmed the chemical integrity of the starch, implying no significant alteration of the molecular structure during extraction and drying.

Three formulations (F1, F2, and F3) of mango seed kernel starch (MSKS) with varying MSKS-to-drug ratios were evaluated for particle size distribution, polydispersity index (PDI), zeta potential, and % encapsulation efficiency (EE). The results highlighted the influence of the MSKS–drug ratios on the physicochemical properties and % encapsulation efficiency of the formulations. The F2 formulation had a higher % encapsulation efficiency and % drug loading. This could be due to a higher amount of starch (polymer) relative to the drug, providing more binding sites and a larger matrix for encapsulating the drug, minimizing drug leakage, and ensuring that more drug is effectively trapped within the nanoparticle matrix.

The reduction in crystal size, confirmed by means of XRD, indicated the transformation of diclofenac sodium from its crystalline form into a more amorphous state within the nanoparticles. The smaller crystal size observed in the MSKSNPs indicates a higher degree of amorphization, which could result in enhanced solubility and dissolution rate [15]. This transformation confirms the successful incorporation of diclofenac into the MSKS nanoparticle system, facilitating improved drug delivery characteristics. TEM results support the successful fabrication of MSKSNPs at the nanoscale, confirming their uniform shape and size distribution. Reduced particle size favors improved drug delivery, as it can enhance the bioavailability, stability, and cellular uptake of the nanoparticles, making them suitable for various pharmaceutical applications [16].

The broad and intense nature of the FTIR absorption peak between 3280–3320 cm^−1^ supports the amorphous nature of the freeze-dried starch in the nanoparticle formulation. Unlike crystalline materials, the presence of such a broad -OH stretching band indicates disordered or non-crystalline regions within the starch, confirming its amorphous state in the MSKSNPs. This structural alteration is essential for enhancing the solubility and dissolution profile of the nanoparticles.

The enhanced epidermal and dermal penetration of diclofenac sodium from MSKSNPs compared to the ethanolic drug solution suggests that the MSKS-based nanoparticle system effectively facilitates transdermal drug delivery. The increased drug retention in skin layers may be attributed to the nanoscale size, which allows for better interaction with the stratum corneum, as well as the hydrophilic–hydrophobic balance provided by the MSKS matrix [17].

The cytotoxicity results revealed that the drug-loaded MSKSNPs had a similar % cell death compared to the drug solution (control). Furthermore, no significant differences were noted among the MSKSNP formulations, underscoring their potential as safe and non-toxic candidates for future applications.

## 4. Materials and Methods

### 4.1. Materials

Mango seed kernel powder was procured from Neoteric, Coimbatore, Tamil Nadu, India. Sodium hydroxide (reagent grade), corn starch (practical grade), trifluoroacetic acid, and phosphate-buffered saline pellets obtained from Sigma-Aldrich, Saint Louis, MO, USA. Diclofenac sodium (USP grade) was procured from Spectrum Chemicals, New Brunswick, NJ, USA. HaCaT human keratinocyte cells were obtained from AddexBio, San Diego, CA, USA. Ethyl alcohol, methyl alcohol, and water (all HPLC grade) were acquired from Sigma-Aldrich, Saint Louis, MO, USA. All remaining reagents employed in this research were of analytical grade.

### 4.2. Isolation and Characterization of Mango Seed Kernel Starch (MSKS)

The mango seed starch was isolated using the procedure mentioned by Shahrim et al., with some modifications that were done in this study [18]. Twenty grams of mango seed kernel powder was dispersed in a 0.5% *w*/*v* sodium hydroxide solution in distilled water with continuous stirring for 6 h. Then, the slurry was filtered using a 200 µm nylon mesh. The residue was washed with distilled water multiple times until a clear filtrate was observed. All the filtrates obtained after washing were mixed and precipitated overnight at 4 °C. The supernatant was removed; the starch slurry was subjected to centrifugation at 3000 rpm for one hour. The sedimented starch was retrieved and dried. A total of 7 batches were prepared, and each batch was dried using different methods of drying, including oven drying and tray drying. The percentage yield was calculated with various process parameters such as the soild–solvent ratio, sedimentation time, and drying method. The percentage yield was calculated using formula:

$$\%Yield=\frac{Amount\;of\;Mango\;seed\;kernel\;starch}{Amount\;of\;Mango\;seed\;kernel\;powder}\times 100$$

The extracted starch was characterized for solubility, pH, moisture content, swelling index, gelatinization temperature, flow properties, and percentage yield, and the data were compared to those of corn starch. The chemical integrity of starch was confirmed using FTIR (Figure 3).

- *pH:* A 20% *w*/*v* dispersion of starch was prepared, and its pH was detected using a pH meter (Mettler-Toledo Seven Compact, Model S220, Columbus, OH, USA), calibrated using standard buffer solutions (pH 4.0, 7.0, and 10.0) before each measurement.
- *Moisture content:* The moisture content was measured by evaporating 5 g of the starch sample by heating it to 105.0 ± 0.1 °C in an oven until the weight became constant. The percentage moisture content was calculated using the following formula:
  $$Moisture\;Content\;\%=\frac{W_{i}-W_{f}}{W_{i}}\times 100$$
  where W_i_ = Sample’s initial weight and W_f_ = final weight of the sample [19].

- *Water Holding Capacity:* A 5% *w*/*v* starch suspension was prepared in a pre-weighed centrifuge tube and centrifuged at 1500 rpm for 5 min. The supernatant was discarded, and the combined weight of the tube and hydrated starch was recorded. Water holding capacity was calculated and expressed as the grams of water retained per 100 g of dry starch. [19].
- *Swelling index:* A 0.1 g starch sample was placed in a centrifuge tube, and 10 mL of distilled water was added. The mixture was heated in a water bath (Julabo GmbH, Seelbach, Germany) at 50.0 ± 0.1 °C for 30 min with continuous shaking. Following heating, the sample was centrifuged at 1500 rpm for 20 min, the supernatant was carefully removed, and the weight of the starch paste was recorded. Swelling power was then calculated using the following formula [20]:

Swelling power = Weight of starch paste/Weight of dry starch sample.

This procedure was carried out over a 50–100 °C temperature range.

- *Gelatinization temperature:* 1 g of MSKS was added to a 20 mL beaker, and 10 mL of deionized water was added. The starch dispersion was subjected to heat at 80 °C on a magnetic heat bench (Benchmark Scientific, Sayreville, NJ, USA). The gelatinization temperature was recorded [21].
- *Amylose: Amylopectin content:* Amylose content of the isolated starch was determined using the method reported by Williams et al. [22]. A 20 mg starch sample was accurately weighed, and 10 mL of 0.5 N KOH was added to prepare a well-mixed suspension. The mixture was then transferred to a 100 mL volumetric flask, and the volume was adjusted to 100 mL with distilled water. Ten milliliters of the resulting starch solution were pipetted into a 50 mL volumetric flask, followed by the addition of 5 mL of 0.1 N HCl and 0.5 mL of iodine reagent. The volume was diluted to 50 mL, and the absorbance (A) was measured at 625 nm. The amylose and amylopectin were determined using the formula below, and their ratio was determined.

Amylose content (%) = (85.24 × A) − 13.19

Amylopectin (%) = 100 − % Amylose

### 4.3. Preparation of Mango Seed Kernel Starch Nanoparticles (MSKSNPs) Loaded with Diclofenac Sodium

Diclofenac-loaded MSKSNPs were formulated using mild alkali hydrolysis followed by ultrasonication using the method described by Ahmad et al. [23]. Three different formulations (F1–F3) were prepared with varying concentrations of MSKS and the drug. A 1.5% *w*/*v* starch suspension was prepared in 0.1 M NaOH and preheated to 80 °C with continuous stirring. The resulting starch solution was subjected to ultrasonication using a probe sonicator (Branson SFX150 Sonifier, St. Louis, MO, USA) operating at 40 kHz for 30 min, with 5-min intervals [24]. Subsequently, the solution was co-precipitated with an ethanolic drug solution in a 1:2 ratio by adding the starch slurry dropwise into the drug solution under constant magnetic stirring. The resulting precipitate was collected by centrifugation at 3000 rpm for 15 min and lyophilized using a freeze dryer (Labconco, Kansas, MO, USA). MSKSNPs were prepared similarly without the drug as a control.

### 4.4. Characterization of Mango Seed Kernel Starch Nanoparticles

#### 4.4.1. Particle Size Distribution and Zeta Potential

Particle size (PDI) and zeta potential were measured using a Malvern Zetasizer Nano ZS (Malvern Panalytical Ltd., Malvern, UK) with a 632.8 nm He-Ne laser. Samples were equilibrated for 3 min in low-volume polystyrene cuvettes (ZEN0118) and analyzed in non-invasive backscatter mode (173°) at 25 °C, with three replicates (N = 3). The freeze-dried samples were redispersed in water, diluted, filtered, and analyzed in the zeta sizer.

#### 4.4.2. Encapsulation Efficiency and Drug Loading

The drug-loaded nanoparticles were dispersed in distilled water and centrifuged at 3700 rpm for 60 min. After separating the supernatant, 1 mL was taken and diluted in 10 mL of distilled water, and the sample was analyzed in HPLC for drug content. The percentage Encapsulation Efficiency (%EE) was calculated using the formula below [11]:

$$\%EE=\frac{Di-Ds}{Di}\times 100,$$

where Di is the initial amount of the drug and Ds is the amount of the drug in the supernatant.

Drug Loading was calculated using the following formula:

$$\%EE=\frac{Di-Ds}{N_{W}}\times 100,$$

where Di is the initial amount of the drug and Ds is the amount of the drug in the supernatant, and $N_{W}$ is the amount of nanoparticles.

HPLC Method: High-performance liquid chromatography (HPLC) analysis was conducted using the method mentioned by Panda et al. by Agilent 1100 Series HPLC system (Agilent Technologies, Santa Clara, CA, USA). The system was equipped with a UV diode array detector (DAD) and operated with HP Chem Station software (Version 32). The mobile phase has 80% methanol and 20% water, containing 0.1% trifluoroacetic acid. This was delivered at a 1.0 mL/min flow rate through an Agilent Eclipse XDB-C18 column (250 mm × 4.6 mm, 5.0 µm particle size). The column temperature was at 25 °C, and the sample injection volume was 20 µL [25]. Diclofenac was quantified with UV detection at a wavelength of 279 nm.

#### 4.4.3. X-ray Diffraction (XRD)

The average crystal size of MSKSNPs was compared with the pure drug diclofenac sodium using a Philips XPERT Powder Diffractometer (Philips Electronic Instruments, Amsterdam, The Netherlands) fitted with a sealed tube Cu anode. The crystallite size of each sample was measured from 10° to 60° two theta using a step increment of 0.1° and a dwell time of 2 s at each step. Drug-loaded MSKSNPs were compared with the orthorhombic phase of diclofenac sodium obtained from the Cambridge Structure Database (refcode = AVASEY, with applied FWHM = 0.5°2θ).

#### 4.4.4. Transmission Electron Microscopy (TEM)

The morphology of MSKSNPs was examined using transmission electron microscopy (TEM). A 10 µL aliquot of the formulation was placed on a copper grid (CF-1.2/1.3, Protochips, Morrisville, NC, USA) and allowed to stand for approximately 1 min. Excess solution was gently blotted with filter paper, followed by the addition of a drop of 1.0% phosphotungstic acid as a stain. After 1 min, the excess stain was removed, and the grid was allowed to dry. Imaging was then performed using a high-contrast TEM (FEI Tecnai G2 Spirit BioTwin CTEM; Mitchel Field, NY, USA).

#### 4.4.5. Fourier Transform Infra-Red Spectrometry (FTIR)

FTIR spectra of mango seed kernel starch and starch nanoparticles were recorded using a Fourier transform infrared (FTIR) spectrometer (Nicolet iS10, Thermo Fisher Scientific, Waltham, MA, USA) equipped with a MicroATR accessory. The spectra were obtained in transmittance mode, averaging 45 scans at a resolution of 4 cm^−1^ over the spectral range of 4000–550 cm^−1^.

### 4.5. In Vitro Release Studies (IVRT)

The in vitro release studies were performed using cellulose acetate membrane (Thermo Fisher^TM^, Bridgewater, NJ, USA), 0.45 µm, 13 mm, hydrophilic on Vertical Franz Diffusion assembly (FDC, Logan, Somerset, NJ, USA). The membrane was placed between the donor and receptor chambers of FDC.A 5 mL of PBS (pH 7.4) were added to the receptor chamber and stirred continuously using a PTFE-coated magnetic bar rotating at 600 rpm. 500 µL of drug-loaded nanoparticles and control (ethanolic drug solution) were applied to the donor chamber. At time points of 1, 2, 3, 4, 5, and 6 h, 300 µL samples of the receptor medium were collected and promptly replaced with an equivalent PBS solution to sustain the sink conditions. The samples were then analyzed using the HPLC method described under Section 4.4.2.

Drug release data from all formulations were analyzed using various kinetic models, including zero-order (cumulative % drug released vs. time), first-order (log cumulative % drug remaining vs. time), and Higuchi (cumulative % drug released vs. square root of time) models. To further elucidate the release mechanism, the data were also fitted to the Korsmeyer–Peppas equation (log cumulative % drug released vs. log time).

### 4.6. In Vitro Permeation Studies (IVPT)

To analyze the permeation of diclofenac sodium in the skin, dermatomed human cadaver skin samples were procured from the posterior torso through the Science Care Skin Bank (Folcroft, PA, USA). Once received, the skin was preserved at −80 °C. On the experiment day, skin samples were defrosted in PBS (pH 7.4) under room temperature for 10 min and mounted on vertical Franz diffusion cells (FDC) (Logan Instruments, Somerset, NJ, USA) with the stratum corneum oriented toward the donor compartment. At same time, the dermal side contacted the receptor compartment.

The solubility of the drug in receptor media (PBS pH 7.4) was tested, and the drug content was analyzed using HPLC to ensure that the drug exhibits requisite solubility in PBS and sink condition is maintained. The receptor was loaded with 5 mL of phosphate-buffered saline (PBS, pH 7.4) and continuously stirred using a PTFE-coated magnetic bar rotating at 600 rpm. The skin temperature was kept at 32 °C by placing the FDC units in a dry heat block (Logan Instruments, Somerset, NJ, USA) set to 32 ± 0.5 °C. Three diffusion cells were utilized for each experimental group (N = 3). After an equilibration period of at least 30 min, aliquot amount (500 µL) of each formulation was spread over to the donor compartment, following the procedure mentioned by Virani et al. [26]. At set time points 2, 4, 6, 8, 12, 16 and 24 h, 500 µL samples of the receptor content were collected and promptly replaced with same amount of fresh buffer solution to maintain sink conditions. The samples were then tested for the drug content using the HPLC method indicated in Section 4.4.2.

The impact of the formulations on skin structural fidelity was assessed by measuring TEWL (Trans-Epidermal Water Loss) before and after applying the formulations to the donor compartment [27]. Cumulative drug permeation was estimated by the below equation:

$$Q_{n}=\frac{C_{n}V_{r}+\sum_{i=0}^{n-1}C_{i}V_{S}}{A}$$

Here, Q_n_ represents the cumulative quantity of drug permeated with respect to area (µg/cm^2^) at various sampling points, C_n_ represents the concentration of drug in the receptor medium at these sampling points (µg/mL). Here, Ci represents the drug concentration in the receptor medium at the previous sampling time i(n − 1)(µg/mL). V_r_ is receptor solution volume (mL), V_s_ is sampling volume (mL), and AA corresponds to the exposed diffusion area of the Franz cell (cm^2^). Here, A = 0.64 cm^2^ (Area of orifice of FDC).

#### Quantification of Drug Content in Skin Samples

After 24 h, the excised skin was carefully removed from the Franz diffusion cells. The area of diffusion was trimmed, and the epidermal and dermal layers were carefully separated using dissecting forceps. Each layer was air-dried, accurately recorded for weight, and placed into Bedbug tubes. To facilitate drug extraction, the skin portions were finely cut with scissors and 1 mL methanol was added to each tube. The samples were homogenized with a BeadBug™ Microtube Homogenizer (D1030, Benchmark Scientific, Sayreville, NJ, USA). The resulting homogenates were centrifuged at 1000 rpm for 5 min and passed through a polypropylene membrane filter (0.45 µm) to discard skin residues. The filtrates were then assessed for drug content using previously described HPLC method, and the quantity of diclofenac sodium retained in the layers of the skin was expressed as µg/mg of skin tissue.

### 4.7. Cytotoxicity Studies

The cytocompatibility of diclofenac sodium–loaded MSKSNPs was evaluated using the HaCaT cell line (human keratinocytes; AddexBio, San Diego, CA, USA) through the Alamar Blue^®^ assay. Cells were plated in a 96-well flat-bottom plate with a density of 10,000 cells/well in 80 µL of 1% FBS-supplemented DMEM and incubated for 18 h. Subsequently, 20 µL of the drug-loaded MSKSNPs and ethanolic solution was incorporated in each cell and incubated for 24 h. Cells treated with only medium served as the negative control. Thereafter, 10 µL of Alamar Blue^®^ reagent (Invitrogen, Carlsbad, CA, USA) was incorporated, followed by incubation period of 3 h. Fluorescence intensity was determined at 540 nm excitation and 590 nm emission with a microplate reader (Tecan, Männedorf, Switzerland). Data presented as the mean ± standard deviation of three independent studies (*n* = 3), with each experiment performed in triplicate. The percentage of cytotoxicity was assessed by the equation:

% Cell Death = 100 − % viability.

Dose–response curves were fitted using a four-parameter logistic (4PL) regression model in Microsoft Excel Solver, with constraints applied for bottom (≥0), top (≤100), Hill slope (≥0), and IC_50_ within experimental concentration range. IC_50_ values were derived as the concentration producing 50% cell death. IC_50_ values were calculated using the formula:

$$Y=Bottom+\frac{Top-Bottom}{1+({\frac{X}{IC50})}^{Hillslope}}.$$

### 4.8. Statistical Analysis

All the studies were carried out in triplicate, with data reported as mean ± SD. Statistical analysis was carried out using two-factor with replication ANOVA, and *p*-values < 0.05 were regarded as statistically significant. Analyses were performed using Microsoft Excel 2021 and Solver version 16.79.1 for cytotoxicity studies.

## 5. Conclusions

Mango seed kernel starch nanoparticles (MSKSNPs) were successfully synthesized using mild alkali hydrolysis and ultrasonication, presenting a simple and efficient method that required less time than conventional acid hydrolysis. Characterization studies revealed that the synthesized MSKSNPs exhibited a more amorphous structure compared to native starch, contributing to their enhanced functionality. The degree of crystallinity of MSKSNPs was found to be 14 nm compared with the pure drug, 33 nm. The cumulative drug release after 24 h for MSKSNPs was found to be 127.46 ± 6.37 µg/cm^2^, compared with that of the ethanolic drug solution, which is 26.56 ± 1.32 µg/cm^2^ with statistical significance of (*p* < 0.001). The amount of diclofenac sodium found in the epidermis was higher for MSKSNPs (5.97 ± 0.30 µg/mg) compared to the ethanolic solution (3.71 ± 0.19 µg/mg) with a statistical significance of (*p* < 0.01), indicating improved skin penetration. Similarly, the drug concentration in the dermis was greater for MSKSNPs (0.82 ± 0.04 µg/mg) than the ethanolic solution (0.21 ± 0.01 µg/mg) with (*p* < 0.001). These nanoparticles demonstrated significantly improved transdermal permeation of diclofenac compared to the ethanolic diclofenac solution at the same concentration (10 mg/mL), underscoring their potential as an efficient vehicle for transdermal drug delivery systems. The cytotoxic studies revealed that the MSKSNPs were safe and non-toxic drug carriers. The four-parameter logistic fit showed IC_50_ values of 9.3 × 10^−5^ µg/mL and 5.9 × 10^−4^ µg/mL for the control and drug-loaded nanoparticles, respectively. This work highlights the value of agro-waste utilization, offering a sustainable approach to developing advanced materials for biomedical applications.

## Figures and Tables

**Table 1:** Effect of solid–solvent ratio, sedimentation time, and method of drying on percentage yield. Each set of studies was conducted in three replicates of three. Data represented as (Mean ± SD).

Sl. No	Drying Method	Soild–Solvent Ratio	Sedimentation Time (h)	% Yield (Mean ± SD)
1	Tray Dryer (40 °C) for 6 h	1:10	24	17.2 ± 2.0
2	Tray Dryer (40 °C) for 6 h	1:10	48	20.2 ± 3.2
3	Tray Dryer (40 °C) for 6 h	1:12	24	15.4 ± 2.4
4	Air dry (RT 20 °C) for 24 h	1:12	24	18.6 ± 2.3
5	Air dry (RT 20 °C) for 24 h	1:12	48	36.2 ± 3.2
6	Freeze drying for 24 h	1:10	48	20.2 ± 2.1
7	Freeze drying for 24 h	1:12	48	30.4 ± 3.2
8	Freeze drying for 24 h	1:14	48	67.7 ± 5.2
9	Freeze drying for 24 h	1:15	48	60.5 ± 3.2

**Table 2:** Physicochemical properties of mango seed kernel starch (MSKS) compared with corn starch. The studies were performed using the methodology indicated in Section 2.2. All of the experiments were conducted in three replicates of three. Results expressed as (Mean ± SD).

Sl. No.	Parameter	Mango Seed Kernel Starch (Mean ± SD)	Corn Starch (Mean ± SD)
1	Solubility (%)	17.0 ± 2.8	14.0 ± 3.2
2	pH	7.0 ± 1.2	7.0 ± 0.6
3	Moisture Content (%)	7.4 ± 0.8	11.8 ± 1.2
4	Water Holding Capacity (%)	79.35 ± 0.8	72.93 ± 0.6
5	Swelling Power (g/g)	3.20 ± 0.16	2.30 ± 0.52
6	Gelatinization Temperature (°C)	60.0 ± 2.5	66 ± 00
7	Amylose/Amylopectin Content	0.35	0.33

**Table 3:** Effect of mango seed kernel starch (MSKS):DRUG ratio on particle size distribution, PdI, zeta potential, %EE, and %DL. All of the experiments were conducted in replicates of three. Results expressed as (Mean ± SD).

Formulation	MSKS:DRUG	Particle Size Distribution (nm) (Mean ± SD)	Polydispersity Index (Mean ± SD)	Zeta Potential (mV) (Mean ± SD)	% Encapsulation Efficiency (EE) (Mean ± SD)	% Drug Loading (DL) (Mean ± SD)
F1	1:1	167.0 ± 1.3	0.34 ± 0.05	−11.00 ± 0.05	83.0 ± 5.0	28.4 ± 3.6
F2	2:1	140.0 ± 3.6	0.42 ± 0.03	−31.20 ± 0.13	92.4 ± 3.7	31.0 ±2.0
F3	1:2	123.0 ± 4.2	0.47 ± 0.02	−27.93 ± 0.14	87.6 ± 3.0	24.0 ±1.4

**Table 4:** In vitro cumulative drug release (%CDR), logarithmic, and square-root time data for diclofenac sodium from drug-loaded nanoparticles and control formulations. Values are represented as the mean ± SD (*n* = 3). % CDR indicates the % of drug released at each time point, while % drug remaining represents the unreleased portion of the total drug. Logarithmic and square-root time transformations were used to evaluate the release kinetics according to the first-order and Higuchi models, respectively.

Drug-Loaded Nanoparticles						
Time (h)	Log Time	SQRT Time	% CDR (M ± SD)	Log % CDR (M ± SD)	% Drug Remaining (M ± SD)	Log % Drug Remaining (M ± SD)
0.00	0.00	0.00	0.00 ± 00	00 ± 00	100.00	2.00
1.00	0.00	1.00	8.81 ± 0.93	0.94 ± 0.04	91.19 ± 0.93	1.96 ± 0.04
2.00	0.30	1.41	14.16 ± 0.65	1.15 ± 0.02	85.84 ± 0.65	1.93 ± 0.02
3.00	0.48	1.73	23.79 ± 0.61	1.38 ± 0.01	76.21 ± 0.61	1.88 ± 0.01
4.00	0.60	2.00	30.80 ± 1.51	1.49 ± 0.02	69.20 ± 1.51	1.84 ± 0.02
5.00	0.70	2.24	37.38 ± 0.93	1.57 ± 0.01	62.62 ± 0.93	1.80 ± 0.01
6.00	0.77	2.45	45.36 ± 1.50	1.66 ± 0.01	54.63 ± 1.50	1.74 ± 0.01
**Control**						
**Time (h)**	**Log Time**	**SQRT** **Time**	**% CDR** **(M** **±** **SD)**	**Log % CDR** **(M** **±** **SD)**	**% Drug Remaining** **(M** **±** **SD)**	**Log % Drug Remaining** **(M** **±** **SD)**
0.00	0.00	0.00	0.00 ± 00	0.00 ± 00	100.00 ± 00	2.00 ± 00
1.00	0.00	1.00	0.84 ± 1.06	−0.08 ± 0.27	99.16 ± 1.06	2.00 ± 0.27
2.00	0.30	1.41	2.53 ± 0.91	0.40 ± 0.12	97.47 ± 0.91	1.99 ± 0.12
3.00	0.48	1.73	4.72 ± 0.48	0.67 ± 0.04	95.28 ± 0.48	1.98 ± 0.04
4.00	0.60	2.00	5.61 ± 0.33	0.75 ± 0.02	94.39 ± 0.33	1.97 ± 0.02
5.00	0.70	2.24	7.51 ± 0.64	0.88 ± 0.03	92.49 ± 0.64	1.97 ± 0.03
6.00	0.78	2.45	8.42 ± 0.91	0.93 ± 0.04	91.58 ± 0.91	1.96 ± 0.04

**Table 5:** Correlation coefficients (R^2^ values) obtained from kinetic model fitting of diclofenac sodium release from drug-loaded nanoparticles and control (ethanolic drug solution). The release values were evaluated using zero-order, Higuchi, first-order, and Korsmeyer–Peppas kinetic models to determine the predominant drug release profile. Higher R^2^ values indicate better model fitting.

Formulation	Zero Order R^2^ Values	Higuchi R^2^ Values	First-Order R^2^ Values	Korsemeyer–Peppas R^2^ Values
Drug-loaded Nanoparticles	0.981	0.995	0.98	0.95
Control (ethanolic drug solution)	0.976	0.994	0.98	0.97

**Table 6:** Dose–response curve fitting parameters for diclofenac sodium in control and nanoparticle (NP) formulations.

Parameter	Control	NP
Bottom	0	0
Top	48	48
Hill Slope	1	1
logIC_50_	2.2	2.2

**Table 7:** Percentage cell death of control (C) and diclofenac-loaded nanoparticles (NP) at varying concentrations (µg/mL). Data shown as the mean ± SD (*n* = 3). Cell death increased in a concentration-dependent manner for both control and NP formulations (*p* > 0.05) for all of the concentrations.

Conc. (µg/mL)	% Death Control	% Death NP
3.9	0	0
7.81	24.25 ± 3.4	28.99 ± 5.2
15.6	27.81 ± 2.8	27.72 ± 4.1
31.25	36.35 ± 3.00	37.71 ± 3.3
62.5	38.71 ± 3.6	42.77 ± 3.4
125	47.92 ± 2.5	47.91 ± 2.7

## Data Availability

The data presented in this study are available on request from the corresponding author due to due to privacy/ethical restrictions or institutional policies.

## References

[1] Shariatinia Z. (2019). Pharmaceutical applications of natural polysaccharides. Natural Polysaccharides in Drug Delivery and Biomedical Applications.

[2] Joseph T.M., Unni A.B., Joshy K., Kar Mahapatra D., Haponiuk J., Thomas S. (2023). Emerging Bio-Based Polymers from Lab to Market: Current Strategies, Market Dynamics and Research Trends. C.

[3] Builders P.F., Arhewoh M.I. (2016). Pharmaceutical applications of native starch in conventional drug delivery. Starch-Stärke.

[4] Vilpoux O.F., Brito V.H., Cereda M.P. (2019). Starch extracted from corms, roots, rhizomes, and tubers for food application. Starches for Food Application.

[5] Bangar S.P., Dhull S.B., Manzoor M., Chandak A., Esua O.J. (2024). Functionality and Applications of Non-Conventional Starches from Different Sources. Starch-Stärke.

[6] Hassan L., Muhammad A., Aliyu R., Idris Z., Izuagie T., Umar K., Sani N. (2013). Extraction and characterisation of starches from four varieties of *Mangifera indica* seeds. IOSR J. Appl. Chem..

[7] Taggart P., Mitchell J. (2009). Starch. Handbook of Hydrocolloids.

[8] Masina N., Choonara Y.E., Kumar P., du Toit L.C., Govender M., Indermun S., Pillay V. (2017). A review of the chemical modification techniques of starch. Carbohydr. Polym..

[9] Kim H.-Y., Park S.S., Lim S.-T. (2015). Preparation, characterization and utilization of starch nanoparticles. Colloids Surf. B Biointerfaces.

[10] Caldonazo A., Almeida S.L., Bonetti A.F., Lazo R.E.L., Mengarda M., Murakami F.S. (2021). Pharmaceutical applications of starch nanoparticles: A scoping review. Int. J. Biol. Macromol..

[11] El-Naggar M.E., El-Rafie M., El-Sheikh M.A., El-Feky G.S., Hebeish A. (2015). Synthesis, characterization, release kinetics and toxicity profile of drug-loaded starch nanoparticles. Int. J. Biol. Macromol..

[12] Chaksmithanont P., Bangsitthideth K., Arunprasert K., Patrojanasophon P., Pornpitchanarong C. (2024). Statistical-Based Optimization of Modified *Mangifera indica* Fruit Starch as Substituent for Pharmaceutical Tableting Excipient. Polymers.

[13] Devi M.G., Santosh K.R., Kusuma A., Pattnaik G., Jal S. (2024). Design, optimization and evaluation of telmisartan fast dissolving film employing mango kernel starch as a new natural super disintegrant. Advancement in Animal Handling and Generative AI for Pre-clinical Studies.

[14] Zhang B., Wang K., Hasjim J., Li E., Flanagan B.M., Gidley M.J., Dhital S. (2014). Freeze-drying changes the structure and digestibility of B-polymorphic starches. J. Agric. Food Chem..

[15] Bates S., Zografi G., Engers D., Morris K., Crowley K., Newman A. (2006). Analysis of amorphous and nanocrystalline solids from their X-ray diffraction patterns. Pharm. Res..

[16] Kulkarni S.A., Feng S.-S. (2013). Effects of particle size and surface modification on cellular uptake and biodistribution of polymeric nanoparticles for drug delivery. Pharm. Res..

[17] Alkilani A.Z., Nasereddin J., Hamed R., Nimrawi S., Hussein G., Abo-Zour H., Donnelly R.F. (2022). Beneath the skin: A review of current trends and future prospects of transdermal drug delivery systems. Pharmaceutics.

[18] Shahrim N., Sarifuddin N., Ismail H. (2018). Extraction and characterization of starch from mango seeds. J. Phys. Conf. Ser..

[19] Omojola M.O., Akinkunmi Y., Olufunsho K., Egharevba H., Martins E. (2010). Isolation and physico-chemical characterization of cola starch. Afr. J. Food Agric. Nutr. Dev..

[20] Daramola B., Osanyinlusi S.A. (2006). Investigation on modification of cassava starch using active components of ginger roots (*Zingiber officinale* Roscoe). Afr. J. Biotechnol..

[21] Attama A., Nnamani P., Mbonu I., Adiku M. (2003). Effect of hypochlorite oxidation on the physicochemical properties of gladiolus starch. J. Pharm. Allied Sci..

[22] Williams P. (1970). A rapid colorimetric procedure for estimating the amylose content of starches and flours. Cereal Chem..

[23] Ahmad M., Gani A., Hassan I., Huang Q., Shabbir H. (2020). Production and characterization of starch nanoparticles by mild alkali hydrolysis and ultra-sonication process. Sci. Rep..

[24] Remanan M.K., Zhu F. (2021). Encapsulation of rutin using quinoa and maize starch nanoparticles. Food Chem..

[25] Panda S.S., Patanaik D., Kumar B.V.R. (2011). New stability-indicating RP-HPLC method for determination of diclofenac potassium and metaxalone from their combined dosage form. Sci. Pharm..

[26] Virani A., Dholaria N., Matharoo N., Michniak-Kohn B. (2023). A study of microemulsion systems for transdermal delivery of risperidone using penetration enhancers. J. Pharm. Sci..

[27] Rath S., Ramanah A., Bon C., Kanfer I. (2020). Application of a dermatopharmacokinetic (DPK) method for bioequivalence assessment of topical metronidazole creams. J. Pharm. Pharm. Sci..

